# An OpenMP-based tool for finding longest common subsequence in bioinformatics

**DOI:** 10.1186/s13104-019-4256-6

**Published:** 2019-04-11

**Authors:** Rayhan Shikder, Parimala Thulasiraman, Pourang Irani, Pingzhao Hu

**Affiliations:** 1 Department of Biochemistry and Medical Genetics and The George and Fay Yee Centre for Healthcare Innovation, University of Manitoba, Room 308-Basic Medical Sciences Building, 745 Bannatyne Avenue, Winnipeg, MB R3E 0J9 Canada; 2 Department of Computer Science, University of Manitoba, Winnipeg, MB Canada; 3 Research Institute in Oncology and Hematology, Winnipeg, MB Canada

**Keywords:** Longest common subsequence (LCS), DNA sequence alignment, Parallel algorithms for LCS, LCS on MPI and OpenMP, Tool for finding LCS

## Abstract

**Objective:**

Finding the longest common subsequence (LCS) among sequences is NP-hard. This is an important problem in bioinformatics for DNA sequence alignment and pattern discovery. In this research, we propose new CPU-based parallel implementations that can provide significant advantages in terms of execution times, monetary cost, and pervasiveness in finding LCS of DNA sequences in an environment where Graphics Processing Units are not available. For general purpose use, we also make the OpenMP-based tool publicly available to end users.

**Result:**

In this study, we develop three novel parallel versions of the LCS algorithm on: (i) distributed memory machine using message passing interface (MPI); (ii) shared memory machine using OpenMP, and (iii) hybrid platform that utilizes both distributed and shared memory using MPI-OpenMP. The experimental results with both simulated and real DNA sequence data show that the shared memory OpenMP implementation provides at least two-times absolute speedup than the best sequential version of the algorithm and a relative speedup of almost 7. We provide a detailed comparison of the execution times among the implementations on different platforms with different versions of the algorithm. We also show that removing branch conditions negatively affects the performance of the CPU-based parallel algorithm on OpenMP platform.

## Introduction

Finding Longest Common Subsequence (LCS) is a classic problem in the field of computer algorithms and has diversified application domains. A subsequence of a string is another string which can be derived from the original string by deleting none or few characters (contiguous or non-contiguous) from the original string. A longest common subsequence of two given strings is a string which is the longest string that is a subsequence of both the strings. The sequential version of the LCS algorithm using “equal-unequal” comparisons takes $\varOmega \left( {\text{mn}} \right)$ time, where m and n represent the length of the two sequences being compared [1, 2]. It is necessary to mention that the problem of finding the LCS of more than two strings is NP-hard in nature [3, 4].

LCS has various applications in multiple fields including DNA sequence alignment in bioinformatics [5–7], speech and image recognition [8, 9], file comparison, optimization of database query etc. [10]. In the field of bioinformatics, pattern discovery helps to discover common patterns among DNA sequences of interest which might suggest that they have biological relation among themselves (e.g., similar biological functions) [11]. In discovering patterns between sequences, LCS plays an important role to find the longest common region between two sequences. Although a praiseworthy amount of efforts have been made in the task of pattern discovery, with the increase of sequence lengths, algorithms seemingly face performance bottlenecks [12]. Furthermore, with the advent of next-generation sequencing technologies, sequence data is increasing rapidly [13], which demands algorithms with minimum possible execution time. Parallel algorithms can play a vital role in this regard.

Out of the parallel solutions of the LCS problem, anti-diagonal [14] and bit-parallel [15] algorithms are few of the firsts and noteworthy attempts. Recently, with the rise of Graphics Processing Unit (GPU)-based accelerators, several Compute Unified Device Architecture (CUDA)-based GPU targeted solutions to the LCS problem have been proposed. Yang et al. [16] are one of the firsts to propose an improved row-wise independent parallel version of the LCS algorithm by changing the data dependency used by a dynamic programming approach and using unique memory-access properties of GPUs. More recently, Li et al. [17] have proposed a parallel formulation of the anti-diagonal approach to the LCS algorithm using a GPU-based model. Although these GPU-based models offer faster execution times, GPU devices are still quite expensive in nature, hence only few computers are equipped with GPUs. In such cases, to achieve performance improvement, CPU-based parallel LCS algorithms (e.g. message passing interface (MPI) and open multi-processing (OpenMP)) are still greatly demanded. However, to the best of our knowledge, there is no such publicly available CPU-based tool for the end users. We addressed this gap by developing a new OpenMP-based tool for the end users by improving the row-wise independent version [16] of the LCS algorithm. Moreover, we also developed two other CPU-based parallel implementations (MPI, hybrid MPI-OpenMP) of the algorithm and provided a detailed benchmarking of all these implementations on simulated and real DNA sequence data, which was absent for this version of the LCS algorithm. The main contributions of this study are listed below.

1. A new OpenMP-based publicly available tool for finding length of LCS of DNA sequences for the end users.
2. A detailed benchmarking of the newly developed CPU-based parallel algorithms using different performance metrics on both simulated and real DNA sequence data, where we found that our OpenMP-based algorithm provides at-least 2 times absolute speedup (compared to the best sequential version) and 7 times relative speedup (compared to using only 1 thread).
3. A comparison of the newly developed OpenMP-based LCS algorithm with and without branch conditions.

## Main text

### Preliminaries

Given two sequence strings $A\left[ {1,2, \ldots , m} \right]$ and $B\left[ {1,2, \ldots , n} \right]$, the LCS of the two strings can be found by calculating the longest common subsequence of all possible prefix strings of $A$ and $B$. The LCS of a prefix pair $A\left[ {1,2, \ldots ,i} \right]$ and $B\left[ {1,2, \ldots ,j} \right]$ can be calculated using the previously calculated prefix pairs with the following recurrence relation:

1 $$R\left[ {i,j} \right] = ~\left\{ {\begin{array}{*{20}{l}} 0 \\ {R\left[ {i - 1,j - 1} \right] + 1} \\ {\text{max}\left( {R\left[ {i - 1,j} \right],R\left[ {i,j - 1} \right]} \right)} \\ \end{array} \begin{array}{*{20}{l}} {if~i = 0~or~j = 0} \\ {if~A\left[ i \right] = B\left[ j \right]} \\ {otherwise} \\ \end{array} } \right.$$

Here, $R$ is a score table consisting of the lengths of the longest common subsequences of all the possible prefixes of the two strings. The length of longest common subsequence of $A$ and $B$ can be found in the cell $R\left[ {m,n} \right]$ of table $R$. From Eq. 1, we can see that the value of a cell $R\left[ {i,j} \right]$ in the scoring table R depends on $R\left[ {i - 1,j - 1} \right]$, $R\left[ {i,j - 1} \right]$ and $R\left[ {i - 1,j} \right] .$

#### Row-wise independent algorithm (Version 1)

Yang et al. [16] has devised a row-wise independent parallel algorithm by removing dependency among the cells of the same row. The modified equation is as follows:

2 $$R\left[ {i,j} \right]\, = \,\left\{ \begin{array}{*{20}{ll}} 0\quad \quad \quad \quad \quad \quad \quad \quad \quad \quad \quad \quad \quad \quad \quad \quad \quad \;\; if\,i = 0 \, or\, j = 0 \\ R\left[ {i - 1,j - 1} \right] + 1\qquad \quad \quad \quad \quad \quad \quad \quad \quad \;\; if\,A\left[ i \right] = B\left[ j \right] \\ { \text{max} }\left( {R\left[ {i - 1,j} \right],R\left[ {i - 1,j - k - 1} \right] + 1} \right)\quad \;\, if\,A = B\left[ {j - k} \right] \\ { \text{max} }\left( {R\left[ {i - 1,j} \right],0} \right)\quad \quad \quad \quad \quad \quad \quad \quad \quad \quad if\,j - k = 0 \\ \end{array} \right.$$

Here, $k$ denotes the number of steps required to find either a match, such as ${\text{A}}\left[ {\text{i}} \right] = {\text{B}}\left[ {{\text{j}} - {\text{k}}} \right]$ or ${\text{j}} - {\text{k}} = 0$. Yang et al. [16] has divided their algorithm into two steps. First, they calculated the values of ${\text{j}} - {\text{k }}$ for every $i$ and stored these values in another table named P. The equation to calculate the value of $P$ is given below.

3 $$P\left[ {i,j} \right]\, = \,\left\{ \begin{array}{l} 0\quad \quad \quad \quad \quad if \, j = 0 \hfill \\ j - 1\quad \quad \quad \;\;if\, B\left[ {j - 1} \right] = C\left[ i \right] \hfill \\ P\left[ {i,j - 1} \right]\quad \;\,otherwise \hfill \\ \end{array} \right.$$

Here, $C$ is the string comprised of the unique characters of string $A$ and string $B$. After that the value of score table $R$ were calculated using the following updated equation.

4 $$R\left[ {i,\,j} \right] = \left\{ {\begin{array}{*{20}ll} 0 & {if\,i = 0\,or\,j = 0} \\ {R\left[ {i - 1,\,j - 1} \right] + 1} & {if\,A\left[ i \right] = B\left[ j \right]} \\ {\max \left( {R\left[ {i - 1,\,j} \right],R\left[ {i - 1,\,j - k - 1} \right] + 1} \right)} & {if\,A = B\left[ {j - k} \right]} \\ {\max \left( {R\left[ {i - 1,\,j} \right],0} \right)} & {if\,j - k = 0} \\ \end{array} } \right.$$

Here, c denotes the index of character $A\left[ {i - 1} \right]$ in string $C$.

#### Row-wise Independent Algorithm (Version 2)

As branching can hamper the performance of parallel algorithms, Yang et al. [16] further modified the calculation of $P$ matrix using the following equation.

5 $$P\left[ {i,j} \right] = \left\{ \begin{array}{l} 0\quad \quad \quad \quad \quad \quad if \, j = 0 \hfill \\ j\quad \quad \quad \quad \quad \quad if\, B\left[ {j - 1} \right] = C\left[ i \right] \hfill \\ P\left[ {i,j - 1} \right]\quad \quad \;otherwise \hfill \\ \end{array} \right.$$

Then Eq. (4) can be rewritten as follows with one branching condition reduced.

6 $$R\left[ {i,j} \right] = ~\left\{ {\begin{array}{*{20}{l}} 0\\ {\max \left( {R\left[ {i - 1,j} \right],0} \right)}\\ {\max (R\left[ {i - 1,j} \right],~R\left[ {i - 1,P\left[ {c,j} \right] - 1} \right] + 1)} \end{array}\begin{array}{*{20}{l}} {if~i = 0~or~j = 0}\\ {if~P\left[ {c,j} \right] = 0}\\ {otherwise} \end{array}} \right.$$

From the two versions of row-wise independent algorithms, we can see that the calculation of values of table *P* only depends on the same row. In contrast, the calculation of the values of score table *R* depends on the previous row only.

### Methodology

For the calculation of the *P* table, each row is independent and can be calculated in a parallel way. Therefore, in our MPI implementation, we scattered the *P* table to all the processes in the beginning. After calculating the corresponding chunk values, process number zero gathers the partial results from all the other processes. For the calculation of score table *R*, elements in each row can be scattered among the processes and gathered afterwards. This scatter and gather operations need to be done for every row. Hence, the communication and synchronization overheads are expected to be higher for the MPI implementation approach.

A shared memory implementation can largely mitigate the communication and synchronization overheads of distributed memory implementations which inspired us to develop the shared memory (OpenMP) implementation. In case of the OpenMP implementation, we used work-sharing construct #pragma omp parallel for (an OpenMP directive for sharing iterations of a loop among the available threads) to compute the elements of a single row of the score table *R* in parallel. We tried different scheduling strategies (static, dynamic, and guided) for sharing works among the threads. The calculation of the P table was also shared among threads. This time, the outer loop was parallelized using #pragma omp parallel for construct, as every row is independent of each other.

In the hybrid MPI-OpenMP approach, we selected the optimum number of processes and threads from the experiments of MPI and OpenMP approach. After that we scattered every row among processes and inside a single process we further shared the chunk of rows among threads using #pragma omp parallel for. To account for longer DNA sequences, we optimized the space complexity of all the three implementations where we kept only the current and the previous row of the score table.

### Results and discussion

#### Data sets and specifications of the computer

We used two different data sets for our experiments. First one is a simulated DNA sequence data, collected from University of California Riverside’s (UCR) random DNA sequence generator [18]. The lengths of the different pairs of sequences are between 128 base pairs to 32,768 base pairs. The second data set consists of 8 virus genome sequence pairs and two entire chromosome genome sequence pairs of two eukaryotes, collected from the website of National Center for Biotechnology Information (NCBI) [19]. The selected sequence lengths vary from 359 base pairs to 32,276 base pairs for the viruses, and from 15,05,371 base pairs to 1,61,99,981 base pairs for the eukaryotes. Table 1 represents the selected virus and eukaryote pairs and their sequence lengths.

**Table 1:** Information of real DNA sequence data sets collected from NCBI [19]

#	Species types	Sequence A	Sequence B
1	Virus	Potato spindle tuber viroid (360 bp)	Tomato apical stunt viroid (359 bp)
2		Rottboellia yellow mottle virus (4194 bp)	Carrot mottle virus (4193 bp)
3		Rehmannia mosaic virus (6395 bp)	Tobacco mosaic virus (6395 bp)
4		Potato virus A (9588 bp)	Soybean mosaic virus N (9585 bp)
5		Chicken megrivirus (9566 bp)	Chicken picornavirus 4 (9564 bp)
6		Microbacterium phage VitulaEligans (17,534 bp)	Rhizoctonia cerealis alphaendornavirus 1 (17,486 bp)
7		Lucheng Rn rat coronavirus (28,763 bp)	Helicobacter phage Pt1918U (28,760 bp)
8		Lactococcus phage ASCC368 (32,276 bp)	Uncultured mediterranean phage uvMED (32,133 bp)
9	Eukaryotes	Athene cunicularia (Chromosome 25, 1,505,370 bp)	Bombus terrestris (Chromosome LG B18, 3,078,061 bp)
10		Athene cunicularia (Chromosome 25, 1,505,370 bp)	Bombus terrestris (Chromosome LG B01, 16,199,981 bp)

*bp* stands for the number base pairs

All the experiments were run on University of Manitoba’s on-campus cluster computing system (Mercury machine). The cluster consists of four fully connected computing nodes with 2-gigabit ethernet lines between every pair of nodes. Each node consists of two 14-core Intel Xeon E5-2680 v4 2.40 GHz CPUs with 128 GB of RAM. Having a total of 28 cores inside, with the help of hyper-threading, each node is capable of running twice as many hardware threads (56 threads) at a time.

#### Comparison among different approaches

**Fig. 1:**
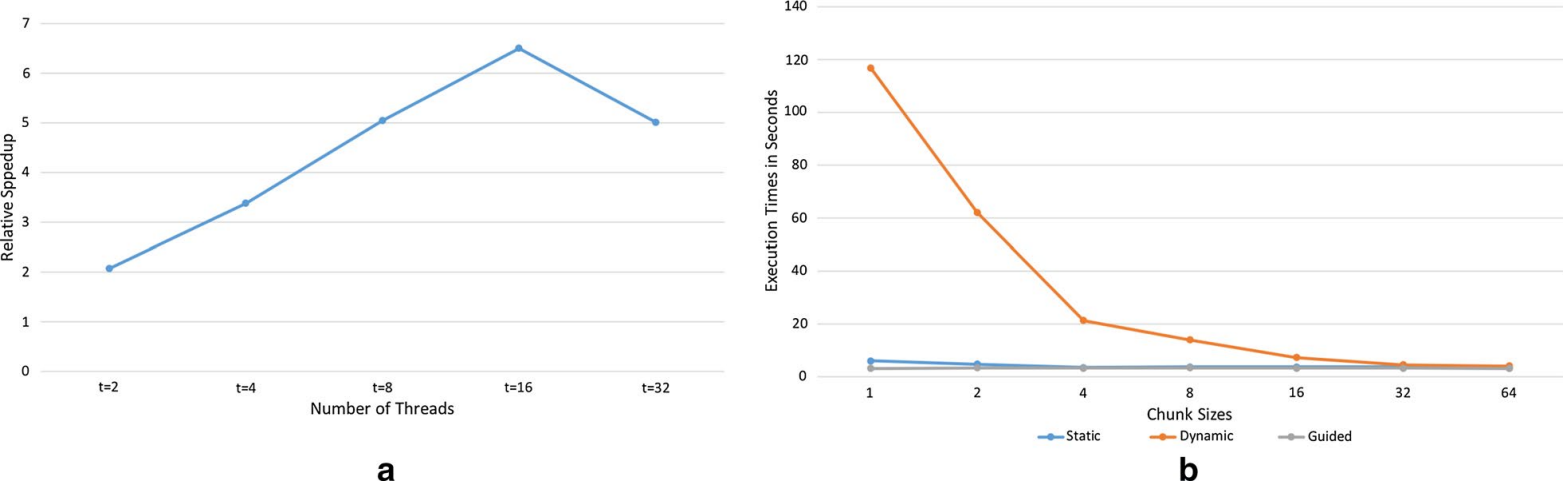
Tuning number of threads and chunk sizes of OpenMP using simulated data. **a** Relative speedup with different number of threads. **b** Execution times (in seconds) for different scheduling strategies and chunk sizes. Number of threads was 16. Sequence lengths were set to 32,768 for both cases

For the MPI approach, we tuned for the number of processes and found that using 4 process gives better relative speedup. For the OpenMP approach, we tuned for the number of threads and the scheduling strategy (static, dynamic, and guided). We found that using 16 threads and a static scheduling of work sharing among the threads provided 7 times relative speedup (see Fig. 1a, b). Finally, for the hybrid MPI-OpenMP approach, we used 4 processes (or nodes) and 16 threads.

**Fig. 2:**
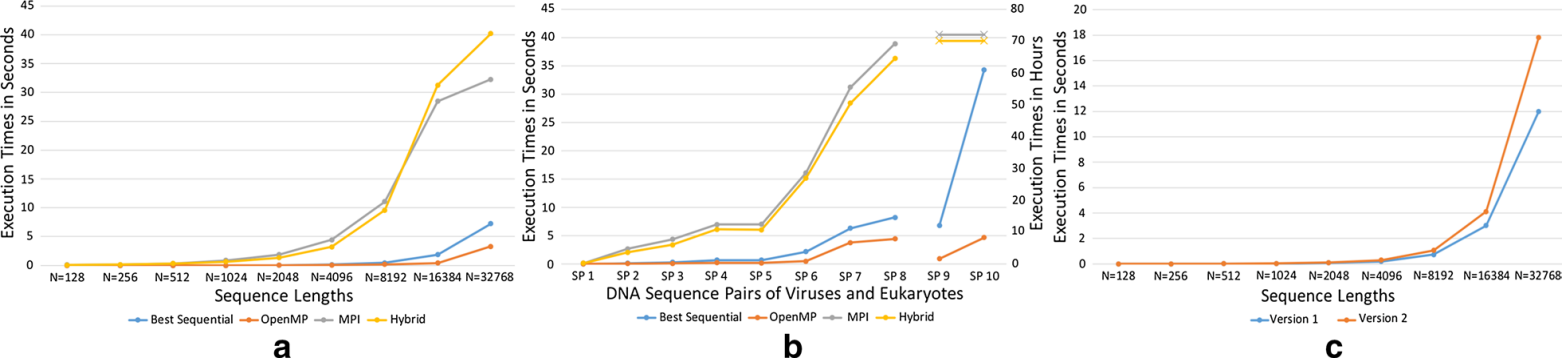
Performance evaluation using both simulated and real data. **a** Execution times for different implementations with varying sequence lengths for the simulated dataset. **b** Execution times for different implementations with different real DNA sequences. Here “SP” stands for sequence pairs from Table 1. The primary (left side) y-axis (execution times in seconds) describes the timing of sequence pairs SP 1 to SP 8 for virus, the secondary (right side) y-axis (execution times in hours) describes the timings of SP 9 and SP 10 for Eukaryotes. Points marked by cross signs denote that those experiments took more than 7 days to complete. **c** Execution times for different lengths of sequence strings from sequential implementation of the two versions of the row-wise independent algorithm

For comparison purpose, we experimented with a varying number of sequence lengths. Figure 2a, illustrates the execution times for different implementations where we can see that our OpenMP implementation outperforms all the other approaches and is almost 2 times faster than the best sequential version. However, the MPI approach provides poor results due to the increased amount of communication and synchronization overhead caused by *m* scatter and gather operations (blocking in nature). The hybrid MPI-OpenMP approach performs the worst. As in the hybrid approach, the number of scatter and gather operations is the same as the MPI approach, and it also adds synchronization overheads of the OpenMP, and therefore this implementation provides the worst result. This observation indicates that distributed memory implementation is discouraged for the LCS algorithm. In order to validate our results, we also experimented with the real-DNA sequence data (see Table 1). From Fig. 2b, we can see that even for the real data the OpenMP implementation is having at-least 2 times speedup from the best sequential version. For longer DNA sequences (SP 9, SP 10 in Fig. 2b), the OpenMP speedups are even higher, whereas the MPI and the hybrid implementations took more than a week to complete.

#### Comparison between the two versions of the algorithm in OpenMP approach

In the above experiments, we used version 2 (without branching) of the row-wise independent algorithm. In order to compare the execution times of the two versions (version 1 and version 2), we also developed the version 1. Figure 2c illustrates the execution times for the two versions with varying sequence sizes and 1 thread only where we can see that version 1 performs relatively better than version 2 of the algorithm. Although version 2 has removed branching conditions, it has added more computations which might be the reason for its relatively bad execution times. Furthermore, CPU architectures are much better at branch predictions than GPUs. Therefore, the second version of the row-wise independent parallel algorithm performed well on GPUs [16] but not on CPUs.

## Limitations

Our study investigated parallelization of the row-wise independent version of the LCS algorithm only, as it provided ease in parallelization using the MPI, and OpenMP frameworks. As we found that the version of the row-wise independent algorithm with branching performs better than the other version, we will investigate this version in more detail in the future. We will also investigate other versions of the algorithm with the goal of finding better parallelization strategies.

### Availability and requirements

Project name: LCS row parallel (CPU)
Project home page: https://github.com/RayhanShikder/lcs_parallel
Operating systems: Platform independent
Programming language: C
Other requirements: gcc 4.8.5 or later, OpenMPI version 1.10.7 or later, OpenMP version 3.1 or later
License: MIT License
Any restrictions to use by non-academics: None.

## Abbreviations

CUDA: compute unified device architecture
GPU: graphics processing unit
LCS: longest common subsequence
MPI: message passing interface
OpenMP: open multi-processing
UCR: University of California Riverside
NCBI: National Centre for Biotechnology Information
